# Supplementary material for: Satisfaction and attrition in the UK healthcare sector over the past decade
Source: PLoS One. 2023 Apr 13;18(4):e0284516. doi: 10.1371/journal.pone.0284516 (PMC10101409; doi:10.1371/journal.pone.0284516)
Supplement: S1 Table — (PDF) [file pone.0284516.s001.pdf]

S1 Table: How the trajectory of job related feelings over time differs in healthcare workers, relative to other public and private sector workers (fixed-effects OLS models)

	Dependent variable:					
	Job feel tense	Job feel uneasy	Job feel worried	Job feel depressed	Job feel gloomy	Job feel miserable
Wave	0.0066	0.0292***	0.0425***	0.0555***	0.0486***	0.0398***
	(0.00919)	(0.00887)	(0.00866)	(0.00801)	(0.00835)	(0.00830)
Non-public sector	0.003	0.0123	-0.0439	0.0753**	0.0203	0.0801**
	(0.0379)	(0.0366)	(0.0357)	(0.0330)	(0.0345)	(0.0342)
Non-public sector x wave	-0.00433	-0.00409	-0.00424	-0.00487	0.00036	-0.00319
	(0.00433)	(0.00417)	(0.00408)	(0.00377)	(0.00393)	(0.00391)
Public sector (excl healthcare)	-0.0334	-0.0334	-0.0819**	0.00321	-0.0272	-0.00551
	(0.0399)	(0.0385)	(0.0376)	(0.0348)	(0.0363)	(0.0360)
Public sector x wave	0.00511	0.00563	0.00567	0.00538	0.00648	0.00691
	(0.00468)	(0.00452)	(0.00441)	(0.00408)	(0.00426)	(0.00423)
Age	0.0164*	0.00264	0.0200**	0.00141	-0.00514	-0.0024
	(0.00854)	(0.00824)	(0.00805)	(0.00745)	(0.00776)	(0.00771)
Age^2	-0.000537***	-0.000368***	-0.000647***	-0.000482***	-0.000427***	-0.000332***
	(5.28e-05)	(5.09e-05)	(4.97e-05)	(4.60e-05)	(4.80e-05)	(4.77e-05)
Married	-0.0283*	-0.00954	0.000369	0.00814	0.0105	0.0126
	(0.0161)	(0.0155)	(0.0152)	(0.0140)	(0.0146)	(0.0145)
# dependent children	-0.0247***	-0.00584	-0.0160**	-0.00935	-0.0151**	-0.0102
	(0.00723)	(0.00698)	(0.00681)	(0.00630)	(0.00657)	(0.00653)
Has degree	0.165***	0.140***	0.125***	0.0798**	0.0631*	0.0388
	(0.0406)	(0.0392)	(0.0383)	(0.0354)	(0.0369)	(0.0367)
Monthly income	2.23e-05***	1.23e-05***	1.17e-05***	-0.00000213	0.00000469	4.95e-06*
	(3.27e-06)	(3.16e-06)	(3.08e-06)	(2.85e-06)	(2.97e-06)	(2.95e-06)
Not self-employed	0.0999***	0.0898***	-0.00106	0.113***	0.133***	0.137***
	(0.0175)	(0.0169)	(0.0165)	(0.0153)	(0.0159)	(0.0158)
Constant	2.518***	2.176***	2.045***	1.931***	2.278***	1.920***
	(0.305)	(0.294)	(0.287)	(0.266)	(0.277)	(0.275)
Observations	107743	107705	107748	107735	107683	107726
R-squared	0.011	0.002	0.004	0.005	0.004	0.003
Number of individuals	40722	40712	40722	40720	40710	40718

Note: OLS standard errors are shown in parentheses. *** p < 0.01, ** p < 0.05, * p < 0.1
