# Supplementary material for: Satisfaction and attrition in the UK healthcare sector over the past decade
Source: PLoS One. 2023 Apr 13;18(4):e0284516. doi: 10.1371/journal.pone.0284516 (PMC10101409; doi:10.1371/journal.pone.0284516)
Supplement: S1 Fig — (PDF) [file pone.0284516.s002.pdf]

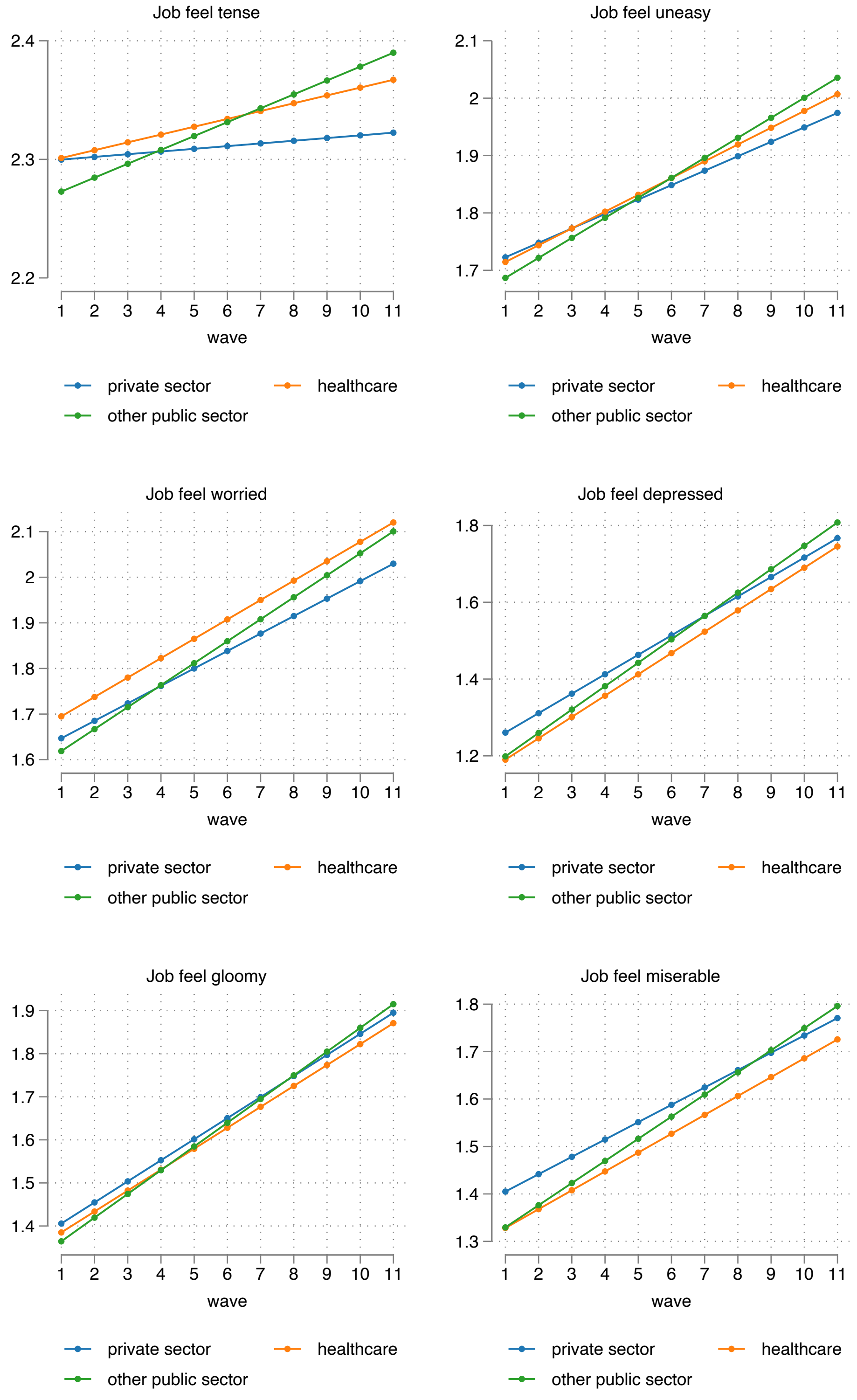

S1 Fig: Estimated job related feelings of people in the healthcare sector over the past decade, relative to other public sector workers, and private sector workers
