# Supplementary material for: Satisfaction and attrition in the UK healthcare sector over the past decade
Source: PLoS One. 2023 Apr 13;18(4):e0284516. doi: 10.1371/journal.pone.0284516 (PMC10101409; doi:10.1371/journal.pone.0284516)
Supplement: Attachment — Submitted filename: Response to reviewers PLoS v1.docx [file pone.0284516.s003.docx]

**Reviewer response for PLoS ONE (PONE-D-22-34427)**

***Satisfaction and attrition in the UK healthcare sector over the past decade***

**Reviewer points (in black) and responses (in blue)**

- The policy recommendations are in a sense obvious, but it is not clear why "systemic changes" are necessary. The authors seem to make a case that more money, more staff, and better management are what the NHS systems need (as they say, the problems they report are evidence of a resource shortage). The fact that they report an uncertain environment is a cause of departures seems to suggest policymakers ought to avoid unspecified systemic change.

Thank you for drawing attention to this source of ambiguity. By ‘systemic changes’, we were trying to draw a distinction between top-down and bottom-up policy in an economic sense. Things like nudges and other workplace well-being interventions (like rest areas or environmental changes) would represent bottom-up measures to tackle, for example, things like poor morale and low mood during shifts. These would be relatively low-cost and address the problem from an individual psychological well-being perspective. However, these interventions can only go so far. They would not have an effect if material conditions were so adverse as to lead to attrition or poor mental well-being. For example, no nudge would be able to compensate for a doctor or nurse that is expected to attend to twice as many patients as would be considered ‘acceptable’. At this point, there are obviously structural causes (e.g. unreasonable working hours, poor workload distribution, low wages) that would need to be addressed. This is what was meant by systemic change, but perhaps the phrasing is not quite appropriate.

‘Brute force’ structural changes would be things like hiring more workers and increasing wages, coupled with regulation that may help with concerns around working hours and family, for example. However, there may be other inefficiencies within the NHS system that could improve workloads by implementing smarter management approaches. In our review of the literature, it seems as though inefficient bureaucracy / poor management are a common complain among healthcare workers.

We agree though that any policy or structural change would need to be clear and transparent rather than unanticipated and destabilising. For a risk averse doctor, one would expect that guarantees about workload and better management (for example) would reduce anxiety.

We have added further clarification to **p26** on the uncertainty point, and to try to make the policy section easier to follow. We have also added a reference to a Lancet report in response to your second point below which may also help to add some justification for policy reforms.

- There is a case for at least some discussion of devolution, which in principle might lead to variation in quality of management, uncertainty, public sector pay, and such. I don't know of the surveys have the right samples to say anything illuminating but the authors might at least discuss the issue. It seems to be the kind of issue for which there is scope to learn useful things across the four UK systems.

Thank you for this suggestion. The NHS workforce statistics only cover England, which does prevent us from comparing leaving reasons across the four devolved countries. The UKHLS is representative of the UK population. Looking at the data 84.3% of the sample is from England, which corresponds very closely to a mid-2021 ONS England to UK population ratio of 84.4%. Meaningful statistical comparisons between England and other UK countries would be difficult due to the small number of healthcare workers outside of England in the sample (we would be left with between 100 and 200 healthcare workers from each of Scotland, Wales, and NI).

However, we agree that it would be worth briefly commenting on the differences between the four countries.

It is generally difficult to compare performance across devolved nations because they do not collect the same statistics (Hawkes, 2013). However, staff to patient ratios seem to be better outside of England. There may be other cultural differences which are harder to quantify in terms of their effects, such as an English emphasis on internal competition rather than the Scottish emphasis on collaboration (Hawkes, 2013). A recent Lancet commissioned report has found some differences in the devolved nations, though again there is still no clear ‘winning system’ (Anderson et al, 2021). However, the report does mention that Scotland seems to have made the most progress towards better workforce planning, which may be likely to remove some of the pressures leading to dissatisfaction and/or attrition. The report also confirms Hawkes’ (2013) observation that staff to patient ratios are generally higher in devolved countries. However, the combined populations of Wales, Scotland, and NI are still about a fifth of England’s population. This is likely to make it easier to adjust staffing levels to meet demand in these three nations. The Lancet report does highlight that England’s competitive system is ‘dysfunctional’, and reforms are likely to be needed there in particular to improve conditions for both staff and patients.

We have briefly discussed some of the above issues in a new paragraph on **p25**.

Hawkes N. How different are NHS systems across the UK since devolution? BMJ 2013; 346 :f3066 doi:10.1136/bmj.f3066

Anderson, M., Pitchforth, E., Asaria, M., Brayne, C., Casadei, B., Charlesworth, A., ... & Mossialos, E. (2021). LSE–Lancet Commission on the future of the NHS: re-laying the foundations for an equitable and efficient health and care service after COVID-19. *The Lancet*, *397*(10288), 1915-1978.

- Otherwise, it's paltry, but I would recommend the authors give it one more read to tune up the prose a bit. The abstract, first page, introduction to the literature review, and concluding sections can be a bit choppy and obscure the motivation and findings on the first or second reading.

Thank you for this suggestion, as well as your general appreciation of the paper. We have re-read the paper and made some changes to the manuscript that will hopefully aid readability. These will be visible via tracked changes, but we note the following main changes:

- the abstract has been reworded slightly to help with clarity
- the first part of the introduction on p3 – p4 has been tweaked to emphasise the fact that there have been two major shocks to the NHS over the last decade, which gives rise to our research question.
- We have added a short paragraph to the beginning of section 1.1 to introduce the two topic areas that our review focuses on before going into the subsections. We also tweaked the final paragraph of the introduction for better coherence.
- Some small tweaks were made to sections 2 and 3 to fix minor grammatical errors and a sentence was removed from p22 to avoid discussion/speculation in the results section.
